# Sol–gel synthesized zinc oxide nanorods and their structural and optical investigation for optoelectronic application

**DOI:** 10.1186/1556-276X-9-429

**Published:** 2014-08-25

**Authors:** Kai Loong Foo, Uda Hashim, Kashif Muhammad, Chun Hong Voon

**Affiliations:** 1 Nano Biochip Research Group, Institute of Nano Electronic Engineering (INEE), Universiti Malaysia Perlis (UniMAP), Kangar, Perlis 01000, Malaysia

**Keywords:** Zinc oxide nanorods, Hydrothermal growth, Solvent, Refractive index, Bandgap

## Abstract

Nanostructured zinc oxide (ZnO) nanorods (NRs) with hexagonal wurtzite structures were synthesized using an easy and low-cost bottom-up hydrothermal growth technique. ZnO thin films were prepared with the use of four different solvents, namely, methanol, ethanol, isopropanol, and 2-methoxyethanol, and then used as seed layer templates for the subsequent growth of the ZnO NRs. The influences of the different solvents on the structural and optical properties were investigated through scanning electron microscopy, X-ray diffraction, Fourier transform infrared spectroscopy, ultraviolet–visible spectroscopy, and photoluminescence. The obtained X-ray diffraction patterns showed that the synthesized ZnO NRs were single crystals and exhibited a preferred orientation along the (002) plane. In addition, the calculated results from the specific models of the refractive index are consistent with the experimental data. The ZnO NRs that grew from the 2-methoxyethanol seeded layer exhibited the smallest grain size (39.18 nm), largest diffracted intensities on the (002) plane, and highest bandgap (3.21 eV).

## Background

Top-down and bottom-up methods are two types of approaches used in nanotechnology and nanofabrication [1]. The bottom-up approach is more advantageous than the top-down approach because the former has a better chance of producing nanostructures with less defects, more homogenous chemical composition, and better short- and long-range ordering [2]. Semiconductor nanorods (NRs) and nanowires possess convenient and useful physical, electrical, and optoelectronic properties, and thus, they are highly suitable for diverse applications [3,4].

ZnO, one of the II-VI semiconductor materials, has attracted considerable interest because of its wide bandgap (approximately 3.37 eV), high exciton binding energy (approximately 60 meV), and long-term stability [5,6]. ZnO has been applied in various applications, such as in light-emitting diode [7], gas and chemical sensors [8-10], ultraviolet (UV) detector [11,12], solar cell [13,14], and biomolecular sensors [15,16]. To create high-quality ZnO NRs, various techniques have been proposed, such as the aqueous hydrothermal growth [10], metal-organic chemical vapor deposition [17], vapor phase epitaxy [18], vapor phase transport [19], and vapor–liquid-solid method [20].

Among these methods, the aqueous hydrothermal technique is an easy and convenient method for the cultivation of ZnO NRs. In addition, this technique had some promising advantages, like its capability for large-scale production at low temperature and the production of epitaxial, anisotropic ZnO NRs [21,22]. By using this method and varying the chemical use, reaction temperature, molarity, and pH of the solution, a variety of ZnO nanostructures can be formed, such as nanowires (NWs) [16,23], nanoflakes [24], nanorods [25], nanobelts [26], and nanotubes [27].

In this study, we demonstrated a low-cost hydrothermal growth method to synthesize ZnO NRs on a Si substrate, with the use of different types of solvents. Moreover, the effects of the solvents on the structural and optical properties were investigated. Studying the solvents is important because this factor remarkably affects the structural and optical properties of the ZnO NRs. To the best of our knowledge, no published literature is available that analyzed the effects of different seeded layers on the structural and optical properties of ZnO NRs. Moreover, a comparison of such NRs with the specific models of the refractive index has not been published.

## Methods

### ZnO seed solution preparation

Homogenous and uniform ZnO nanoparticles were deposited using the sol–gel spin coating method [28]. Before seed layer deposition, the ZnO solution was prepared using zinc acetate dihydrate [Zn (CH_3_COO)_2_ · 2H_2_O] as a precursor and monoethanolamine (MEA) as a stabilizer. In this study, methanol (MeOH), ethanol (EtOH), isopropanol (IPA), and 2-methoxyethanol (2-ME) were used as solvents. All of the chemicals were used without further purification. ZnO sol (0.2 M) was obtained by mixing 4.4 g of zinc acetate dihydrate with 100 ml of solvent. To ensure that the zinc powder was completely dissolved in the solvent, the mixed solution was stirred on a hot plate at 60°C for 20 min. Then, 1.2216 g of MEA was gradually added to the ZnO solution, while stirring constantly at 60°C for 2 h. The milky solution was then changed into a homogenous and transparent ZnO solution. The solution was stored for 24 h to age at room temperature (RT) before deposition.

### ZnO seed layer preparation

**Figure 1:**
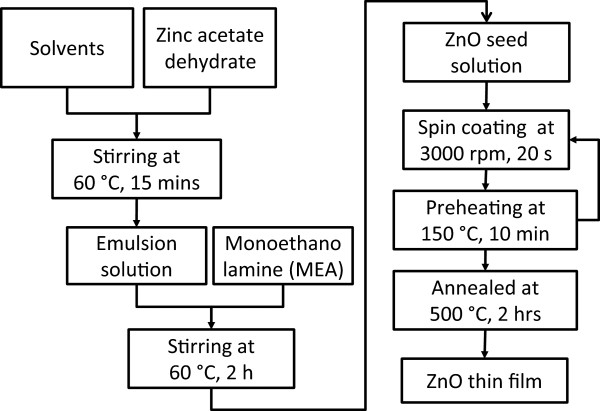
ZnO thin film preparation process flow.

In this experiment, a p-type Si (100) wafer was used as the substrate. Prior to the ZnO seed layer deposition process, the substrate underwent standard cleaning processes, in which it was ultrasonically cleaned with hydrochloric acid, acetone, and isopropanol. The native oxide on the substrate was removed using a buffered oxide etch solution, and then, the substrate was rinsed with deionized water (DIW). Subsequently, a conventional photoresist spin coater was used to deposit the aged ZnO solution on the cleaned substrates at 3,000 rpm for 20 s. A drying process was then performed on a hot plate at 150°C for 10 min. The same coating process was repeated thrice to obtain thicker and more homogenous ZnO films. The coated films were annealed at 500°C for 2 h to remove the organic component and solvent from the films. The annealing process was conducted in the conventional furnace. The preparation of the ZnO thin films is shown in Figure 1.

### ZnO NRs formation

**Figure 2:**
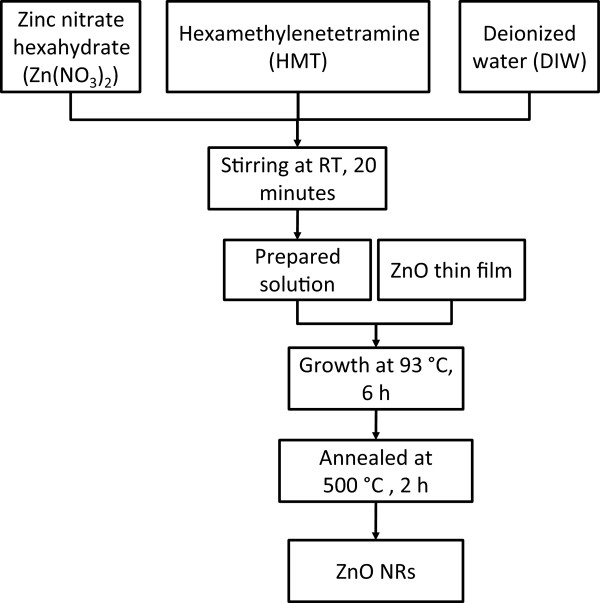
ZnO NR growth process.

After the uniform coating of the ZnO nanoparticles on the substrate, the ZnO NRs were obtained through hydrothermal growth. The growth solution consisted of an aqueous solution of zinc nitrate hexahydrate, which acted as the Zn^2+^ source, and hexamethylenetetramine (HMT). The concentration of the Zn (NO_3_)_2_ was maintained at 35 mM, and the molar ratio of the Zn (NO_3_)_2_ to HMT was 1:1. For the complete dissolution of the Zn (NO_3_)_2_ and HMT powder in DIW, the resultant solution was stirred using a magnetic stirrer for 20 min at RT. The ZnO NRs were grown by immersing the substrate with the seeded layer that was placed upside down in the prepared aqueous solution. During the growth process, the aqueous solution was heated at 93°C for 6 h in a regular laboratory oven. After the growth process, the samples were thoroughly rinsed with DIW to eliminate the residual salts from the surface of the samples and then dried with a blower. Finally, the ZnO NRs on the Si substrate were heat-treated at 500°C for 2 h. The growth process of the ZnO NRs is presented in Figure 2.

### Material characterization

The surface morphology of the ZnO NRs was analyzed using scanning electron microscopy (SEM, Hitachi SU-70, Hitachi, Ltd, Minato-ku, Japan). X-ray diffraction (XRD, Bruker D8, Bruker AXS, Inc., Madison, WI, USA) with a Cu Kα radiation (*λ* = 1.54 Ǻ) was used to study the crystallization and structural properties of the NRs. The absorbed chemical compounds that exited on the surface of the ZnO NRs and SiO_2_/Si substrate were identified using the Fourier transform infrared spectroscopy (FTIR, PerkinElmer Spectrum 400 spectrometer, PerkinElmer, Waltham, MA, USA). A UV-visible-near-infrared spectrophotometer from PerkinElmer was used to study the optical properties of the ZnO NRs at RT. In addition, the optical and luminescence properties of the ZnO NRs were studied through photoluminescence (PL, Horiba Fluorolog-3 for PL spectroscopy, HORIBA Jobin Yvon Inc., USA).

## Results and discussion

### SEM characterization

**Figure 3:**
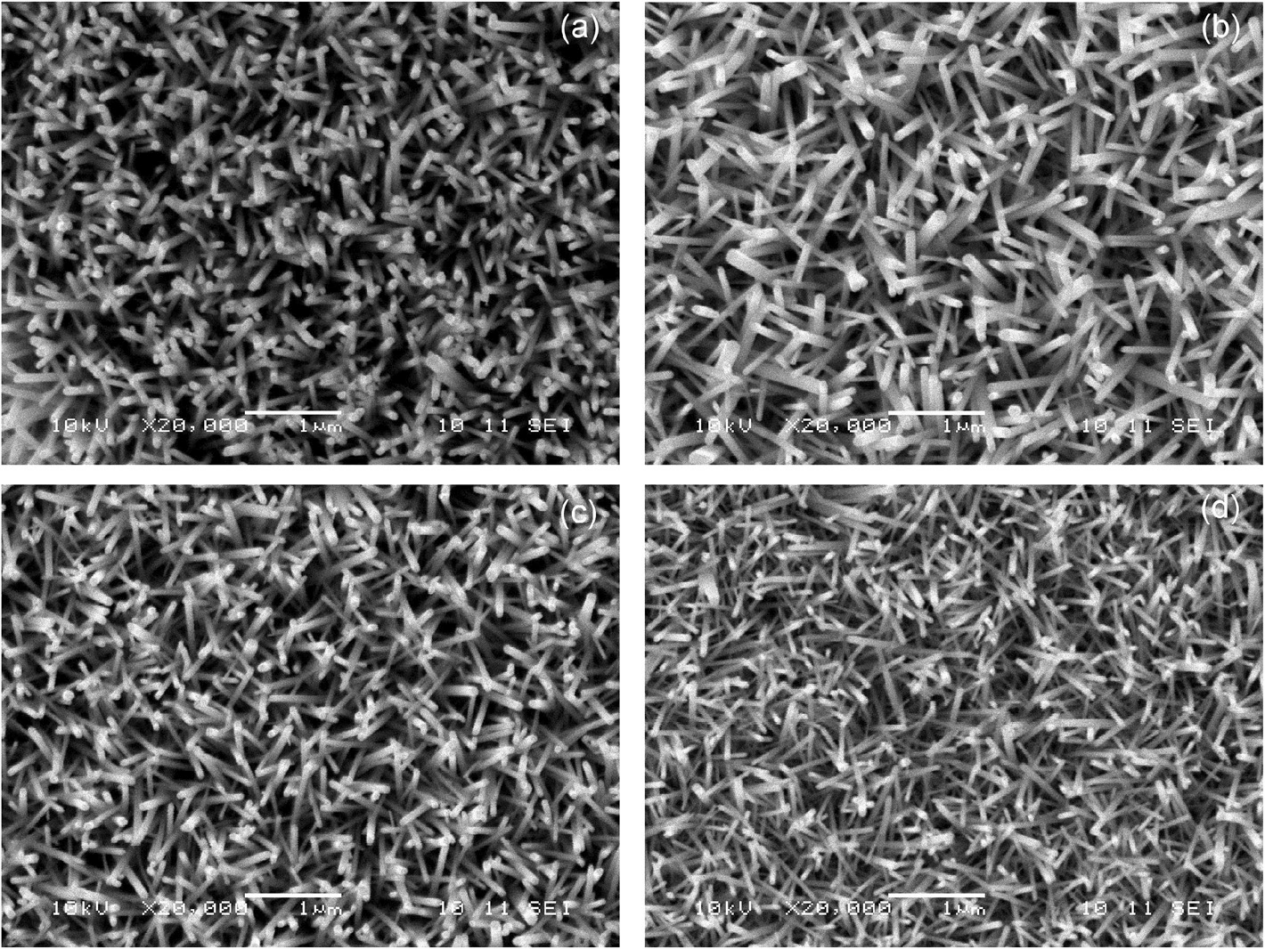
SEM images of ZnO NRs prepared with different solvents: (a) MeOH, (b) EtOH, (c) IPA, and (d) 2-ME.

The top-view SEM images of the ZnO NRs that were synthesized with the use of different solvents are shown in Figure 3. All of the synthesized ZnO NRs showed a hexagonal-faceted morphology. The diameter of the obtained ZnO NRs was approximately 20 to 50 nm. The NRs covered the entire surface of the substrate, and most of these NRs grew into an unchain-like and branched structure. On the basis of the SEM images, the utilization of different solvents evidently resulted in different diameters of the synthesized ZnO NRs. The ZnO NRs that were synthesized using 2-ME provided the smallest diameter, whereas those synthesized with EtOH displayed the largest diameters. The size of the ZnO NRs in diameter is strongly dependent on the grain size of the ZnO seed layer [29]. As the grain size of the seed layer increases, larger sizes of ZnO NRs in diameter are produced.

### XRD characterization

**Figure 4:**
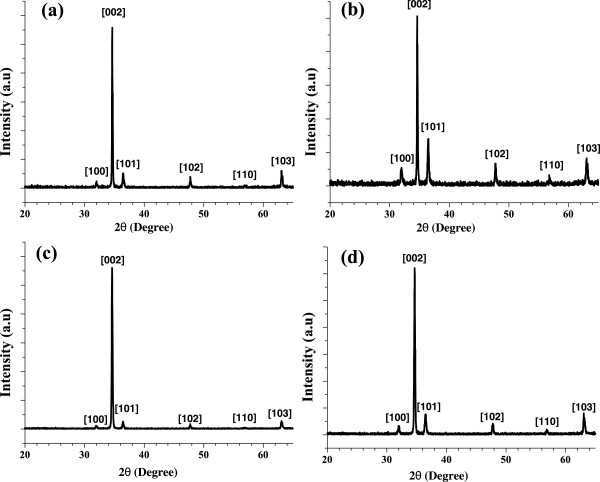
X-ray diffraction patterns of ZnO NRs with hydrothermal growth process: (a) MeOH, (b) EtOH, (c) IPA, and (d) 2-ME.

The crystal structure and microstructure of the as-synthesized ZnO NRs were studied through XRD. Figure 4 shows the XRD patterns of the ZnO NRs that were synthesized on the silicon substrate with the aqueous solutions and different seeded layers. All of the diffraction peaks are consistent with the standard card Joint Committee on Powder Diffraction Standards (JCPDS) 36–1451. The peak intensities were measured in the range of 30° to 70° at 2*θ*. The result showed that the ZnO NRs that were prepared through the hydrothermal growth method presented a remarkably strong diffraction peak at the (002) plane, which is located between 34.5° and 34.6° [30,31]. This finding indicated that all of the ZnO samples possessed pure hexagonal wurtzite structures with high *c*-axis orientations.

Among the peaks, the ZnO NRs that were prepared with EtOH resulted in the narrowest peak of full width at half maximum (FWHM). By contrast, the ZnO NRs that were prepared with 2-ME showed the largest peak of FWHM. Simultaneously, the 2-ME solvent also showed the highest peak intensities on the (002) plane. Compared with the standard diffraction peaks of ZnO, the clear and sharp peaks indicated that the ZnO NRs possessed an excellent crystal quality, with no other diffraction peaks and characteristic peaks of impurities in the ZnO NRs. Therefore, all of the diffraction peaks were similar to those of the bulk ZnO. Table 1 shows the ZnO XRD data from the JCPDS card compared with the measured ZnO XRD results.

**Table 1:** XRD parameters of ZnO NRs

**hkl**	**2** ***θ *** **(°)**				**JCPDS**
	**Observed**				
	**MeOH**	**EtOH**	**IPA**	**2-ME**	
100	32.02	31.98	31.98	32.10	31.76
002	34.52	34.62	34.64	34.68	34.42
101	36.46	36.52	36.5	36.58	36.25
102	47.76	47.8	47.74	47.8	47.53
110	56.94	56.78	56.96	56.86	56.60
103	63.08	63.06	63.08	63.06	62.86

The average grain size of the ZnO NRs was estimated using Scherrer’s formula [32]:

(1) $$D\;=\;\frac{\kappa \lambda }{\mathrm{FWHM}\;\cos\theta }$$

where *κ* is the Scherrer constant, which is dependent on the crystallite shape and can be considered as 0.9 [33,34]; *λ* is the X-ray wavelength of the incident Cu K_α_ radiation, which is 0.154056 nm [35]; FWHM is the full width at half maximum of the respective peak; and *θ* represents the diffraction peak angle. Given that all of the ZnO NRs that were grown through the hydrothermal method exhibited the largest diffraction peaks at the (002) plane, the grain size of the ZnO was calculated along this plane. The calculated crystallite size is presented in Table 2. The result showed that the ZnO NRs that were synthesized on the 2-ME seeded layer produced the smallest crystallite size of 39.18 nm. This result is consistent with the SEM images. However, the largest crystallite size of 58.75 nm was observed when the ZnO NRs were synthesized on the seeded EtOH layer. This finding may be due to the higher viscosity of the EtOH solvent than those of the other solvents.

**Table 2:** Measured structural properties of ZnO NRs using XRD for different solvents

**Solvent**	**XRD (100) peak position**	**XRD (002) peak position**	***a *** **(Ǻ) (100)**	***c *** **(Ǻ) (002)**	**Grain size (nm)**
MeOH	32.02	34.52	3.225	5.192	54.84
EtOH	31.98	34.62	3.229	5.178	58.75
IPA	31.98	34.64	3.229	5.175	45.70
2-ME	32.10	34.68	3.217	5.169	39.18

The lattice constants *a* and *c* of the ZnO wurtzite structure can be calculated using Bragg's law [36]:

(2) $$a\;=\;\sqrt{\frac{1}{3}}\;\frac{\lambda }{\sin\theta }$$

(3) $$c\;=\;\frac{\lambda }{\sin\theta }$$

where *λ* is the X-ray wavelength of the incident Cu Kα radiation (0.154056 nm). For the bulk ZnO from the JCPDS data with card number 36–1451, the pure lattice constants *a* and *c* are 3.2498 and 5.2066 Å, respectively. Based on the results shown in Table 2, all of the ZnO NRs had lower lattice constant values compared with the bulk ZnO. The ZnO NRs prepared with MeOH (*a* = 3.23877 Ǻ and *c* = 5.20987 Ǻ) were closest to the bulk ZnO. This phenomenon can be attributed to the high-temperature annealing condition. Similar results were observed by Lupan et al. [37], in which the increase in temperature decreases the lattice constant of ZnO.

### FTIR characterization

**Figure 5:**
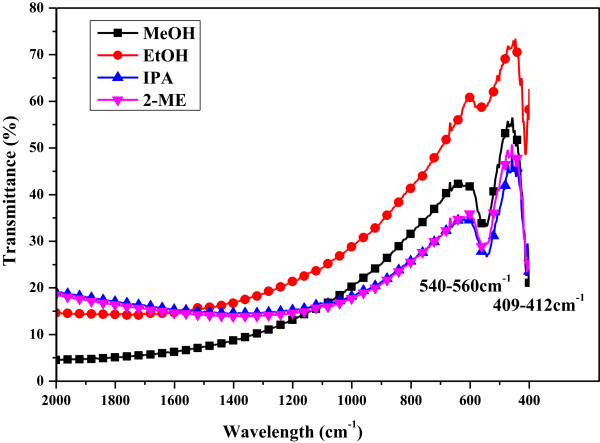
FTIR absorption spectrum of ZnO NRs using various solvents.

Figure 5 illustrates the FTIR spectra of the as-deposited four representative ZnO NRs prepared using four different solvents. Given that the wavelength of the fingerprint of the material ranged from 400 to 2,000 cm^-1^[38], the absorption region was fixed in this region. Overall, the spectrum showed two significant peaks and all of the ZnO NRs that were prepared using different solvents exhibited the same peaks. The ZnO NR morphologies that are grown via wet chemical synthesis prefer the *c*-axis growth [39]. Thus, the ZnO NRs usually had a reference spectrum at around 406 cm^-1^[40]. However, this absorption spectra is found at 410, 412, 409, and 410 cm^-1^ for the ZnO NRs prepared with the use of MeOH, EtOH, IPA, and 2-ME solvents, respectively, because these solvents caused a blueshift in the spectra of as-prepared ZnO NRs. The band from 540 to 560 cm^-1^ is also a stretching mode that is correlated with the ZnO [41,42].

### UV–vis characterization

**Figure 6:**
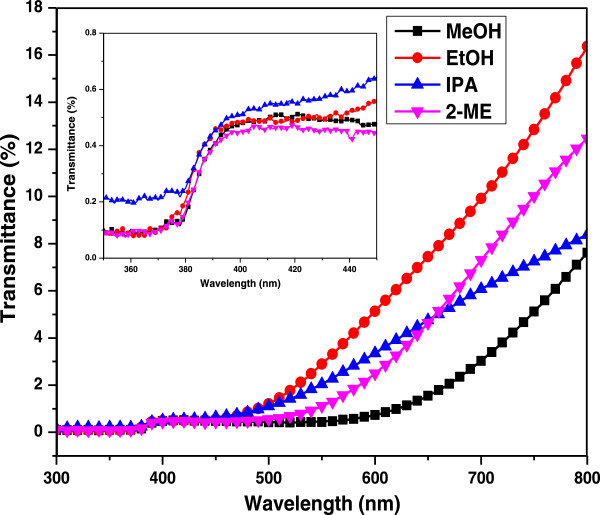
Optical transmittance spectra of hydrothermal derived ZnO NRs.

The transmittance spectra and optical properties of the ZnO NRs in the wavelength range of 300 to 800 nm were investigated through UV-visible spectroscopy at RT. The UV-visible transmittance spectra of the ZnO NRs are shown in Figure 6. The inset of Figure 6 shows the magnified view of transmittance spectrum in the wavelength range of 350 to 450 nm. The results showed that all of the ZnO NRs that were prepared using different solvents exhibited strong excitonic absorption peaks at 378 nm. These peaks indicated that the grown ZnO NRs possessed good optical quality and large exciton binding energy.

**Figure 7:**
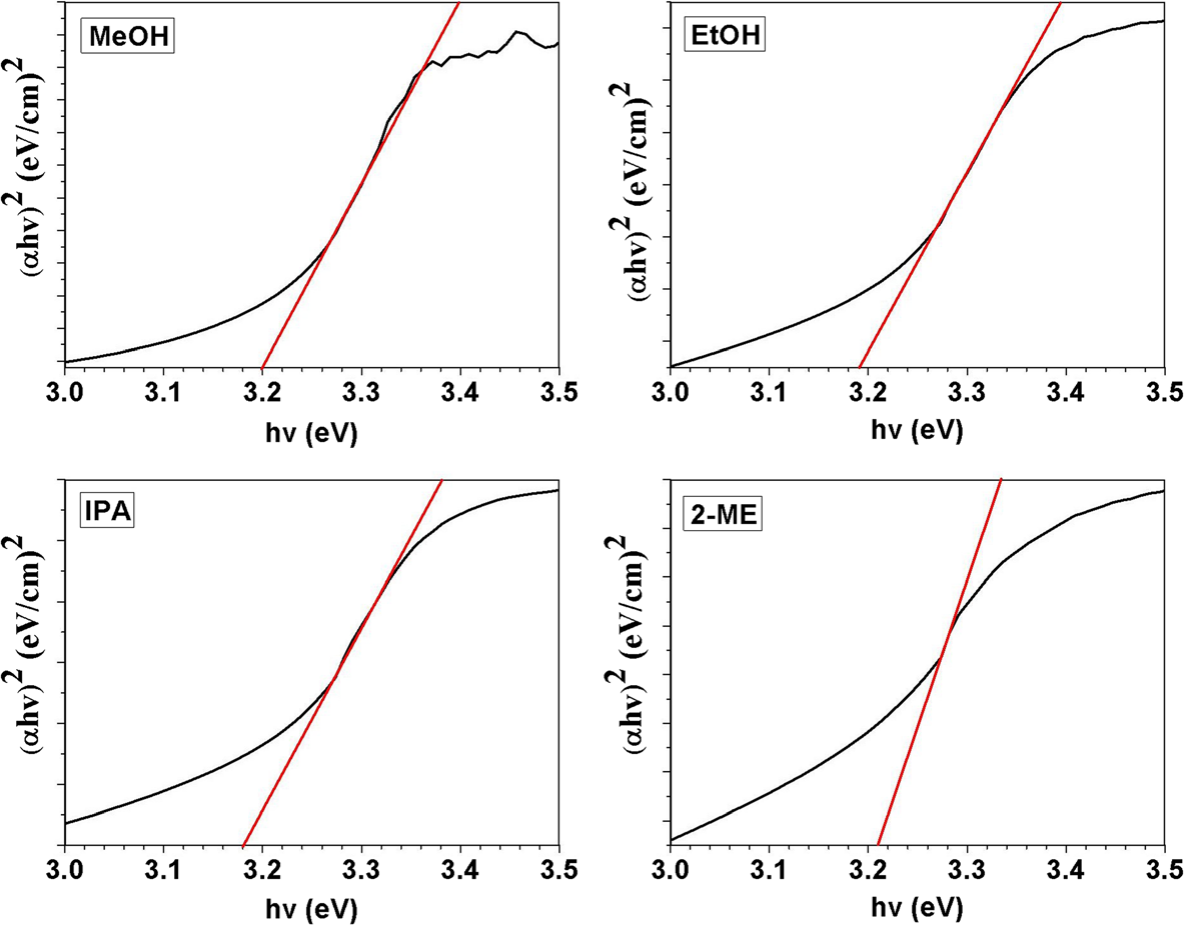
**Plot of ( *α *** **hv) **^**2 **^**versus the photon energy for different solvent derived ZnO thin films.**

The absorption coefficient (*α*) for the direct transition of the ZnO NRs was studied using Equation 4 [43]:

(4) $$\alpha \;=\;\frac{\ln\left(1/T\right)}{d}$$

where *T* is the transmittance of the ZnO films, and *d* is the film thickness. The optical bandgap (*α*hv) dependence on the absorption coefficient (*α*) over the energy range of 3 to 3.5 eV at RT was calculated using the following relation [44]:

(5) $$\alpha \mathrm{hv}\;=\;B{\left(\mathrm{hv}\;-\;E_{g}\right)}^{n}$$

where hv is the photon energy, *B* is the constant, *E*_g_ is the bandgap energy, and *n* is the allowed direct band with the value of ½. The direct bandgap energies for the different solvents used were determined by plotting the corresponding Tauc graphs, that is, (*α*hv)^2^ versus hv curves. This method was used to measure the energy difference between the valence and conduction bands. The direct bandgap of the ZnO films was the interception between the tangent to the linear portion of the curve and the hv-axis (Figure 7). The optical bandgaps determined from the curves are summarized in Table 3. The results indicated that the ZnO NRs that were grown with 2-ME for the seed layer preparation showed the highest bandgap (3.21 eV), whereas those grown with the IPA exhibited the lowest bandgap (3.18 eV), which is believed to possess a better conductivity. According to the corresponding bandgap energy (*E*_g_) and absorption band edge (*λ*) of the bulk ZnO, that is, 367 nm and 3.36 eV, respectively [45], the as-grown ZnO NRs possessed a significantly lower bandgap or exhibited a redshift of *E*_g_ from 0.15 to 0.18 eV. This shift can be attributed to the optical confinement effect of the formation of ZnO NRs [46] and the size of the ZnO NRs [47].

**Table 3:** Direct bandgap, calculated refractive indices of ZnO NRs corresponding to optical dielectric constant

**Solvent**	**Bandgap (eV)**			**Refractive index ( *n *)**			**Optical constant (**Ɛ_***∞***_**)**		
MeOH	3.20	3.28^a^	3.25^b^	2.064^i^	2.290^j^	2.329^k^	4.260^i^	5.246^j^	5.426^k^
EtOH	3.19	3.31^c^	3.10^d^	2.070^i^	2.293^j^	2.331^k^	4.286^i^	5.259^j^	5.436^k^
IPA	3.18	3.29^e^	3.27^f^	2.076^i^	2.296^j^	2.334^k^	4.311^i^	5.272^j^	5.445^k^
2-ME	3.21	3.28^g^	3.39^h^	2.058^i^	2.288^j^	2.327^k^	4.235^i^	5.233^j^	5.417^k^

^a^Yi et al*.*[64].

^b^Cao et al. [58].

^c^Karami et al. [59].

^d^Gowthaman et al. [60].

^e^Shakti et al. [61].

^f^Mejía-García et al. [62].

^g^Kashif et al. [23].

^h^Abdullah et al. [63].

^i^Ravindra et al. [51].

^j^Herve and Vandamme [52].

^k^Ghosh et al. [53].

Many attempts have been made to relate the refractive index (*n*) and *E*_g_ through simple relationships [48-51]. However, these relationships of *n* are independent of the temperature and incident photon energy. Herein, the various relationships between *n* and *E*_g_ were reviewed. Ravindra et al. [51] presented a linear form of *n* as a function of *E*_g_:

(6) $$n\;=\;\alpha \;+\;\beta E_{g}$$

where *α* = 4.048 eV^-1^ and *β* = -0.62 eV^-1^. Moreover, light refraction and dispersion were inspired. Herve and Vandamme [52] proposed an empirical relation as follows:

(7) $$n\;=\;\sqrt{1\;+\;{\left(\frac{A}{E_{g}\;+\;B}\right)}^{2}}$$

where *A* = 13.6 eV and *B* = 3.4 eV. For group IV semiconductors, Ghosh et al. [53] published an empirical relationship based on the band structure and quantum dielectric considerations of Penn [54] and Van Vechten [55]:

(8) $$n^{2}\;-\;1\;=\;\frac{A}{{\left(E_{g}\;+\;B\right)}^{2}}$$

where *A* = 25 *E*_g_ + 212, *B* = 0.21 *E*_g_ +4.25, and (*E*_g_ + *B*) refer to an appropriate average *E*_g_ of the material. The calculated refractive indices of the end-point compounds and *E*_g_ are listed in Table 3. In addition, the relation Ɛ_*∞*_ = *n*^2^[56] was used to calculate the optical dielectric constant Ɛ_*∞*_. Our calculated refractive index values are consistent with the experimental values [23,57-63], as shown in Table 3. Therefore, Herve and Vandamme model is an appropriate model for solar cell applications.

### PL characterization

**Figure 8:**
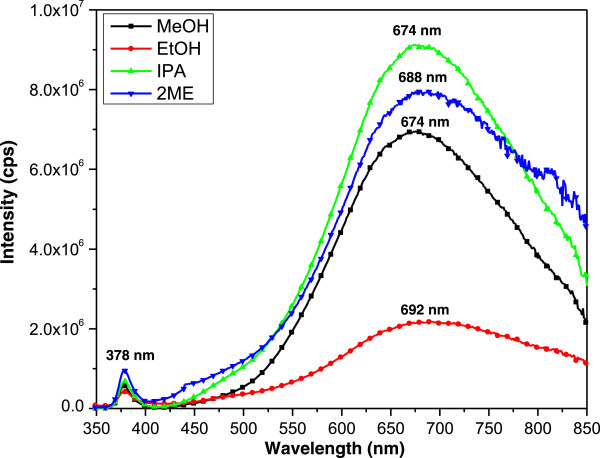
PL spectrum of ZnO NRs grown on different seeded substrate.

The effects of solvents on the luminescence properties of ZnO NRs were studied via PL spectroscopy, with excitation of a xenon lamp at 325 nm. Figure 8 shows the typical spectra for the photoluminescence of ZnO NRs that were grown on different seeded substrates. All the samples demonstrated two dominant peaks, which had UV emissions of 300 to 400 nm and visible emissions at 400 to 800 nm. The first emission band that was located in that UV range was caused by the recombination of free excitons through an exciton-exciton collision process [24,64,65]. In addition, the second emission band, which was a broad intense of green emission, originated from the deep-level emission. This band revealed the radiative recombination of the photogenerated hole with the electrons that belonged to the singly ionized oxygen vacancies [66-68].

UV luminescence can be used to evaluate the crystal quality of a material, whereas visible luminescence can be used to determine structural defects [69]. A study by Abdulgafour [70]. indicates that a higher ratio of UV/visible is an indicative index of a better crystal quality. In the current study, the UV/visible ratios for the ZnO NRs prepared with the use of IPA, MeOH, 2-ME, and EtOH were 13.34, 12.15, 8.32, and 5.14, respectively. Therefore, the UV/visible ratio trend confirms the improvements in crystal quality of the ZnO NRs that were prepared using different solvents.

## Conclusions

In this study, ZnO NRs with a highly crystalline structure were synthesized via a low-cost and convenient hydrothermal technique. The SEM images of the samples demonstrated that the diameters of the hydrothermally synthesized ZnO NRs range from 20 to 50 nm. The XRD patterns exhibited that all of the ZnO NRs had remarkably excellent crystal qualities and high *c*-axis orientations. The calculated bandgap values of the synthesized ZnO NRs were lower than that of the bulk ZnO. The crystal qualities, grain size, diameter, and optical bandgap of the ZnO NRs were affected by the type of solvent used in the ZnO seed layer preparation. The ZnO NRs that were synthesized with the use of 2-ME, a solvent, exhibited the most improved results, in terms of structural and optical properties; these ZnO NRs showed the smallest grain size, smallest crystallite size, and highest bandgap values. The method developed in this study provides a simple and low-cost approach to fabricate ZnO NRs with the desired properties.

## Competing interests

The authors declare that they have no competing interests.

## Authors’ contributions

KLF conducted the sample fabrication and took part in the ZnO NR preparation and characterization and manuscript preparation. UH initialized the research work and coordinated and supervised this team’s work. MK carried out the ZnO NR preparation and characterization. CHV conducted the ZnO NR characterization and manuscript preparation. All authors read and approved the final manuscript.
